# Phenotypic variation from waterlogging in multiple perennial ryegrass varieties under climate change conditions

**DOI:** 10.3389/fpls.2022.954478

**Published:** 2022-08-04

**Authors:** Carl A. Frisk, Georgianna Xistris-Songpanya, Matthieu Osborne, Yastika Biswas, Rainer Melzer, Jon M. Yearsley

**Affiliations:** ^1^School of Biology and Environmental Science, University College Dublin, Dublin, Ireland; ^2^Earth Institute, University College Dublin, Dublin, Ireland

**Keywords:** anthocyanins, chamber experiment, colors, flooding, grasslands, harvest, image analysis, *Lolium perenne*

## Abstract

Identifying how various components of climate change will influence ecosystems and vegetation subsistence will be fundamental to mitigate negative effects. Climate change-induced waterlogging is understudied in comparison to temperature and CO_2_. Grasslands are especially vulnerable through the connection with global food security, with perennial ryegrass dominating many flood-prone pasturelands in North-western Europe. We investigated the effect of long-term waterlogging on phenotypic responses of perennial ryegrass using four common varieties (one diploid and three tetraploid) grown in atmospherically controlled growth chambers during two months of peak growth. The climate treatments compare ambient climatological conditions in North-western Europe to the RCP8.5 climate change scenario in 2050 (+2°C and 550 ppm CO_2_). At the end of each month multiple phenotypic plant measurements were made, the plants were harvested and then allowed to grow back. Using image analysis and principal component analysis (PCA) methodologies, we assessed how multiple predictors (phenotypic, environmental, genotypic, and temporal) influenced overall plant performance, productivity and phenotypic responses. Long-term waterlogging was found to reduce leaf-color intensity, with younger plants having purple hues indicative of anthocyanins. Plant performance and yield was lower in waterlogged plants, with tetraploid varieties coping better than the diploid one. The climate change treatment was found to reduce color intensities further. Flooding was found to reduce plant productivity via reductions in color pigments and root proliferation. These effects will have negative consequences for global food security brought on by increased frequency of extreme weather events and flooding. Our imaging analysis approach to estimate effects of waterlogging can be incorporated into plant health diagnostics tools via remote sensing and drone-technology.

## Introduction

Predicting plant responses caused by climate change is a fundamental challenge that will increasingly impact coming generations (Parmesan and Hanley, 2015). Identifying how the various components of climate change are likely to affect plant responses will inform options of optimal mitigation strategies to minimize any negative effects (Canadell and Raupach, 2008; Pareek et al., 2020). While this is a complex issue affecting all ecosystems, it is especially important for grassland ecosystems due to the unavoidable connection with global food security through agriculture, crop lands and pasture lands (Tester and Langridge, 2010; Kipling et al., 2016a; Lecerf et al., 2019; Raza et al., 2019). There is a growing concern that climate change will result in ecological transformation of grasslands (Kipling et al., 2016b; Wang et al., 2018) which will alter plant phenology (Jentsch et al., 2009; Munson and Long, 2017), biodiversity (Bellocchi and Picon-Cochard, 2021), and productivity (Goliński et al., 2018; Qi et al., 2018).

Grasslands are defined as ecosystems dominated by the Poaceae (grass) taxonomic family, with the ecology varying widely depending on species composition, edaphic factors, topography, management, and climate. Previous studies have suggested that elevated CO_2_ levels are likely to increase photosynthetic capability in grasses, increasing Net Primary Production (NPP) and thereby total yield (Ergon et al., 2018; Yiotis et al., 2021). Meanwhile, higher temperatures are likely to extend the length of the growth season, providing longer time for sustained growth (Höglind et al., 2013; Pembleton et al., 2020). While increased ambient temperatures and elevated CO_2_ act long-term and are the main components of many climate models (e.g., Thornley and Cannell, 1997; Huntingford et al., 2013), altered precipitation regimes are equally important, and predicted to act both short- and long-term (Dore, 2005; Cullen et al., 2009; Brown et al., 2019). Previous grass research from altered precipitation regimes has focused on the effects of prolonged droughts, reduced water-availability and increased desertification (Farfan-Vignolo and Asard, 2012; Cullen et al., 2014; Bothe et al., 2018; Buttler et al., 2019; Yates et al., 2019), however many areas might see the opposite effect, leading to increased flooding (Kiely, 1999; Rosenzweig et al., 2002).

Climate change-induced alterations to precipitation regimes are expected to contribute to increased frequency of extreme weather events, such as severe storms and extreme flooding (Easterling et al., 2000; Semmler and Jacob, 2004). This is partly due to warmer temperatures increasing evaporation, and warmer air being able to hold more moisture, increasing the total amount of water vapor in atmospheric circulation (Hu et al., 2000; O’Gorman and Muller, 2010). Additionally, predicted changes to the precipitation seasonality are likely to change the frequency and distribution of rain events, potentially enhancing the drought-flooding dichotomy (Feng et al., 2013; Kumar, 2013).

Perennial ryegrass (*Lolium perenne*) is a common cool season pasture grass grown extensively throughout its native Eurasian-range and cultivated worldwide due to its high nutritional quality and palatability for livestock (Hunt and Easton, 1989; Hannaway et al., 1999; Smit et al., 2005; Minneé et al., 2019; Tubritt et al., 2020). Many genetically different varieties of perennial ryegrass are bred and cultivated to match the climatological conditions of specific target regions, which in turn causes differences in plant health and yield depending on local environmental suitability (Grogan and Gilliland, 2011; Helgadóttir et al., 2018). Varieties suitable in current conditions under regular precipitation regimes might prove unsuitable under periods of increased flooding (Mustroph, 2018). Flooding can cause long-term waterlogging, affecting overall plant health and total yield (McFarlane et al., 2003; Striker, 2012) and grassland ecosystem function (Fay et al., 2008). It could also impact plant morphology due to phenotypic plasticity (Münzbergová et al., 2017; Mizutani and Kanaoka, 2018). Previous studies have shown that plants can survive the unfavorable conditions but alters their phenotype in the process as a response, with some varieties of the same species being differentially susceptible to the stressor (Song, 2009; Tong et al., 2021; Stasnik et al., 2022). Decrease in growth due to an altered phenotypic response can have devastating impacts on global food security due to the bottom-up reliance of agricultural system productivity from grassland areas (Hazell and Wood, 2008; Baldos and Hertel, 2014). In addition, economic consequences would be especially severe for countries like Ireland and the United Kingdom with large land areas consisting of ryegrass dominated pasture lands and many rivers currently prone and predicted in the future to flood (Blöschl et al., 2019).

Here, we quantify the effects from waterlogging on perennial ryegrass performance and plant health in the light of climate change. We hypothesized that waterlogging would decrease perennial ryegrass performance and lower plant yield, and that the climate change treated plants would perform better under waterlogging than the ambiently treated plants. We further explored whether waterlogging and climate change impacted the phenotypic plasticity of the plants. We investigated this using atmospherically controlled growth chambers and multiple commercial high-producing perennial ryegrass varieties with varying genetic backgrounds in an image analysis framework.

## Materials and methods

### Experimental setup

This study used four atmospherically controlled CONVIRON BDW40 walk-in growth chambers located in the PÈAC (Programme for Experimental Atmospheres and Climate) facility in Rosemount Environmental Research Station belonging to the University College Dublin in Dublin, Ireland. This facility has been used in previous studies to investigate plant responses to elevated CO_2_ (Batke et al., 2018; Yiotis et al., 2021) and atmospheric paleoclimatic reconstruction (Evans-Fitz. Gerald et al., 2016; Porter et al., 2017; Yiotis et al., 2017). Two chambers were chosen to represent typical North-western European climatological conditions (CO_2_-levels at 415 ppm and ambient temperature conditions) while two chambers were chosen to represent the predicted 2050 combined climate change climatological conditions according to the RCP8.5 scenario [elevated CO_2_-levels (550 ppm) and a 2°C increase in temperature (IPCC, 2014)]. The growth chamber climatological baselines (ambient conditions) were constructed from the last thirty years of meteorological data (1989–2018) collated from the meteorological station located at Cork Airport and publicly accessible via the Irish Meteorological Services (Met Éireann). The entire experiment simulated conditions from May to September but only climatological conditions replicating two months of optimal pasture growth (June and July) (McHugh et al., 2020) were used to investigate perennial ryegrass responses to waterlogging (Table 1).

**TABLE 1:** Experimental Growth Chamber setup for the two ambient and the two climate chambers simulating the months June and July in North-western Europe.

SM	DAS	Length	Treatment	Environmental Conditions					
	
		Days		CO_2_	Light/Dark	Max Light Intensity	Day Temp	Night Temp	Humidity
				(ppm)	Hours (h)	(μmol⋅m^–2^⋅s^–1^)	(°C)	(°C)	(%)
June	44–72	28	Ambient	415	17/7	600	14	11	80
	44–72	28	eCO_2_ + 2°C	550	17/7	600	16	13	80
July	73–101	28	Ambient	415	16/8	600	16	13	80
	73–101	28	eCO_2_ + 2°C	550	16/8	600	18	15	80

The conditions in the four chambers were changed as each month ended. Dawn and dusk conditions are included in the light hours. SM, simulated months; DAS, days after sowing.

Four common internationally grown varieties of perennial ryegrass were used for the experiment: *Aberchoice, Abergain, Carraig*, and *Dunluce* (European Commision, 2019). The varieties vary in heading date and ploidy (Table 2), which allowed for an intra-species comparison to identify whether genetic factors might contribute to the response to waterlogging and climate change. At the simulated start of May each chamber was populated with 80 PVC cores (50 cm × 16 cm (ϕ)) filled with John Innes No2 compost (320 cores in total for the four chambers), with the John Innes No2 (Westland) being a loam-mixture compost with peat, horticultural grit, and added nutrients. Each core was sealed inside a plastic bag to allow half of the cores to be waterlogged further on. The 50 cm tall cores allowed for the simulation of largely natural grassland root depth (Wedderburn et al., 2010; Cougnon et al., 2017). Each core was sown with ten seeds from one of the four varieties (DAS 0, Days after sowing) and allowed to germinate. At DAS 43, the most centrally germinated seedling was kept, and the other germinated seeds discarded. The seedlings were then cut to a height of 5 cm to simulate equal growth between all replicates. Waterlogging was initiated in 40 of the 80 cores per chamber on DAS 48, with the additional watering being slowly initiated over three days to reach a stable water level 2 cm above the soil. Non-waterlogged cores continued to be watered normally, once to twice weekly to keep soil moisture around 25%. Waterlogging was actively enforced for one month and then allowed to dissipate naturally. Waterlogging experiments in grasses tend to last around 15 days (e.g., Liu and Jiang, 2015; de la Cruz Jiménez et al., 2017; Ploschuk et al., 2017), with few experiments lasting up to 30 days (McFarlane et al., 2003). The longer duration was implemented due to the increased chance (and consequences) of climate changed-induced extreme flooding events (Dore, 2005; Trenberth, 2011). Chamber treatment, waterlogging, and variety placement were stratified equally among the chambers and then randomized within chambers. The stratification allowed for equal numbers of each variety in each chamber, with half of the 80 cores per chamber being waterlogged for equal comparison for all factors.

**TABLE 2:** Perennial ryegrass (*Lolium perenne*) varieties grown and water status for all chambers and variety replicates.

Variety	Heading	Ploidy	Water Status		
			Logged	Normal	Total
Aberchoice	Late	Diploid	40	40	80
Abergain	Late	Tetraploid	40	40	80
Carraig	Intermediate	Tetraploid	40	40	80
Dunluce	Intermediate	Tetraploid	40	40	80

Each water status is equally divided between all four chambers.

### Phenotypic data collection

Multiple sets of phenotypic data were collected each month to identify the effects on plant performance from the waterlogging. Soil and Plant Analyzer Development (SPAD) readings were conducted using a SPAD-502 Plus Chlorophyll Meter (Konica Minolta) to sample leaf chlorophyll content (e.g., Bothe et al., 2018; Dong et al., 2019). Three representative leaves were sampled from each plant, with each leaf being sampled at three places and averaged for a reliable measurement. Soil moisture was measured at 10 cm depth using a HH2 Moisture Meter with a calibrated WET sensor type WET-2 attachment (Delta-T Devices). At the end of each month (DAS 72, June and 101, July) the maximum height of the plants were measured. After being measured, the plants were harvested by cutting the grasses using scissors at 5 cm above the soil-level, leaving 5 cm of basal grass tissue intact, simulating natural grazing. The plants were then allowed to recover, grow and then be harvested again the subsequent month. The harvested material from each plant was placed on a flat white background and photographed using a high-resolution LUMIX DC-G9 camera (Panasonic) with an accompanied ColorChecker Classic chart (X-rite). This enabled the harvested material to be processed using color-corrective image analysis techniques to identify differences in leaf color hues (e.g., Hu et al., 2013; Li et al., 2014; Zhang et al., 2014; de la Cruz Jiménez et al., 2017). The leaves were photographed immediately after harvest to prevent structural and color degradation (Løkke et al., 2013; Yamauchi and Watada, 2019). After the photographs all harvest material from each plant was oven dried at 65°C for one week and then weighed to measure dried biomass per plant.

### Image analysis

The first stage of image analysis was to convert the color filter array (CFA) in each RAW image from the LUMIX DC-G9 camera into a color-corrected red-green-blue (RGB) image. This was done using a standardized pipeline with the following nine steps: (1) subtract a black value from all pixels in the CFA, (2) set negative pixel values to zero, (3) divide all pixels by the maximum pixel value, (4) correct the white balance by scaling red and blue pixels relative to green pixels in the CFA, (5) convert pixels to unsigned 16-bit integers, demosaiced the CFA into a true-color image using a gradient-corrected linear interpolation (Malvar et al., 2004), (6) transform the image from the camera’s color space to RGB, (7) identify the 24 colors on the X-Rite ColorChecker Classic chart within the image, (8) estimate an affine color-transformation matrix that minimizes the sum of squared deviations between the RGB colors in the image to the known colors of the 24 colored squares, (9) apply the color-transformation matrix to produce a color-correct RGB image. The camera’s image metadata was used to obtain values for black level, white-balance correction and camera color space to RGB conversion.

The second stage of the image analysis extracted RGB values from pixels that corresponded to ryegrass leaves. Ryegrass leaves were placed upon a flat, white, rectangular background which enabled the image to be cropped to the white background. The cropped image was then converted into an HSV color-space and an initial mask created with hue values in the range 0.0–0.4 and 0.875–1.0 (corresponding to yellow, green, and red hues), saturation in the range 0.2–1.0 and value in the range 0.0–0.9. Regions of the mask with connected components containing fewer than 100 pixels were removed before the mask was refined using 50 iterations of an active contours region growing algorithm and saturation values retained if they were in the range 0.25–1.0. The final mask was used to extract the position of pixels in the mask and their RGB values. All image processing was performed using MatLAB (version R2021a.) and its image processing toolbox (Mathworks, 2021).

### Statistical analyses

The ryegrass leaf RGB values from the image analysis were further processed by calculating the median value for each hue from each image of the harvested ryegrass. Median values were used due to their robust statistical properties against outliers and skewed distributions (Chen, 1998; von Hippel, 2005). To dimensionally reduce the three hues into one variable the hues were analyzed using principal component analysis (PCA) from the R package *vegan* (Oksanen et al., 2020). Although variable standardization is normally recommended (Jollife and Cadima, 2016), the hues were analyzed without scaling and centering to preserve the relative values (Lever et al., 2017). We did not expect the removal of scaling to have a detrimental effect on model fitting due to the three bands having similar variances. The principal components (PC) were first tested for normality using the Shapiro-Wilks test (Shapiro and Wilk, 1965) and then analyzed using Kendall’s tau rank correlation (Kendall, 1938) and Wilcoxon’s signed rank test (Wilcoxon, 1945) to test if there were any correlations and differences in mean values between the harvest groups (DAS 72 and 101) and between each water status. Kendall’s tau was used due to the higher robustness and efficiency compared to the otherwise commonly used Spearman’s rho (Spearman, 1904; Croux and Dehon, 2010). The first principal component was further used as a response variable to build a linear model to identify relevant covariates responsible for the combined hues. A similar approach has previously been used by Golzarian and Frick (2011) to investigate early growth stages of grasses. Multiple predictor variables were used to build the linear regression model: phenotypic (dried biomass, maximum height and SPAD measurements as a proxy for chlorophyll content), environmental (soil moisture as a proxy for waterlogging and chamber treatment as proxy for ambient and climate change climatological conditions), genetic (variety as a proxy for ploidy and heading date) and temporal (progression of the season and recovery from the waterlogging). Although mixed-models are generally recommended in this type of hierarchical ecological design (Piepho et al., 2003) the estimation of random effects from a low number of levels is very similar to fitting fixed effects models, and can in some cases cause worse model fits due to zero variance estimates (Harrison et al., 2018). The model terms were subsequently analyzed using a Type II ANOVA (Langsrud, 2003; Smith and Cribbie, 2014). Model selection was then performed to analyze how removing variables in the full model would impact the AIC values and model understanding (Bozdogan, 1987; Aho et al., 2014). All statistical analyses were performed in the statistical software R (version 4.1.3.) (R Core Team, 2022).

## Results

### Plant appearance

**FIGURE 1:**
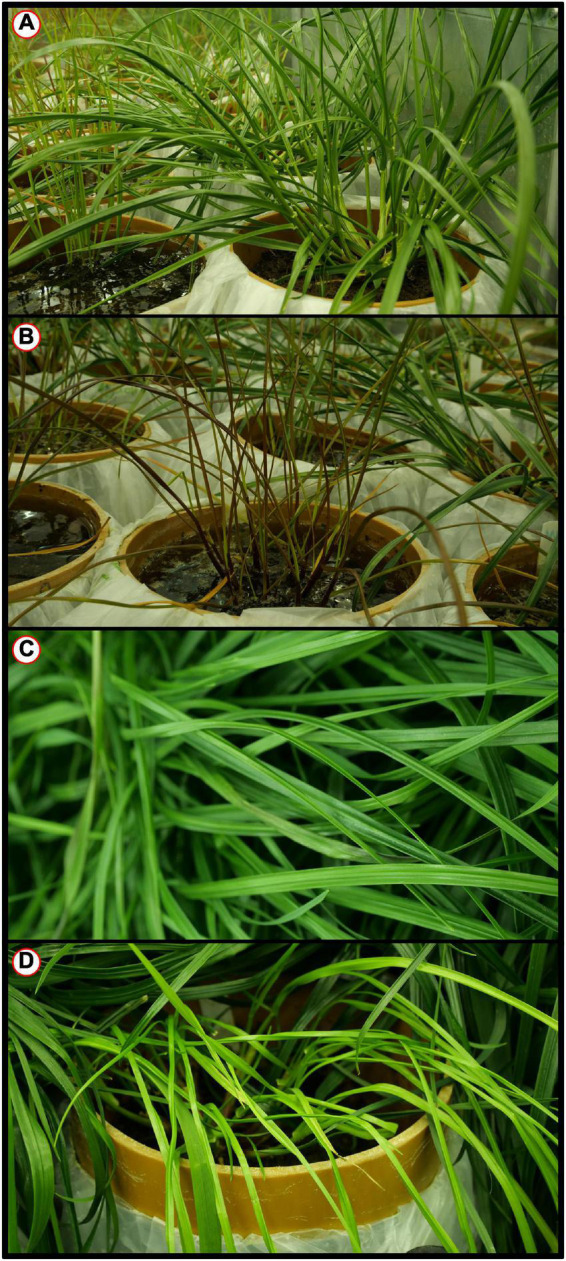
Perennial Ryegrass (*Lolium perenne*) whole plant appearance examples images for selected harvests and water status. **(A)** DAS 72 non-waterlogged. **(B)** DAS 72 waterlogged. **(C)** DAS 101 non-waterlogged. **(D)** DAS 101 waterlogged. Examples images are not color corrected.

**FIGURE 2:**
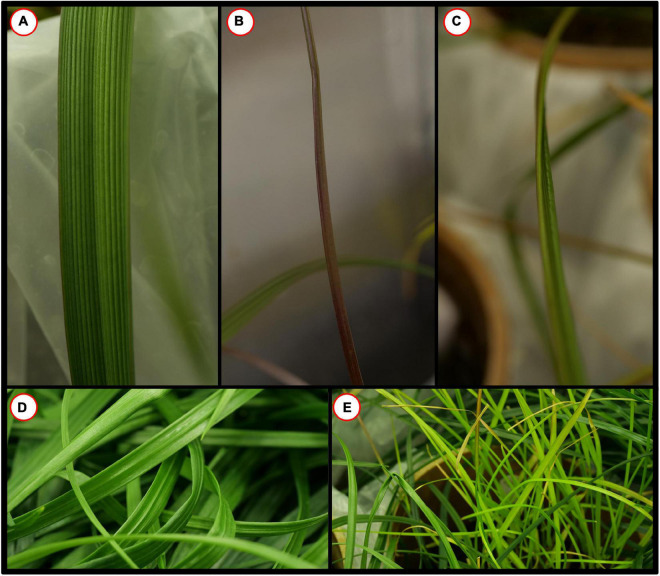
Perennial Ryegrass (*Lolium perenne*) leaf morphology example images for selected harvests and water status. **(A)** DAS 72 non-waterlogged. **(B)** DAS 72 waterlogged. **(C)** DAS 72 waterlogged. **(D)** DAS 101 non-waterlogged. **(E)** DAS 101 waterlogged. Examples images are not color corrected.

**FIGURE 3:**
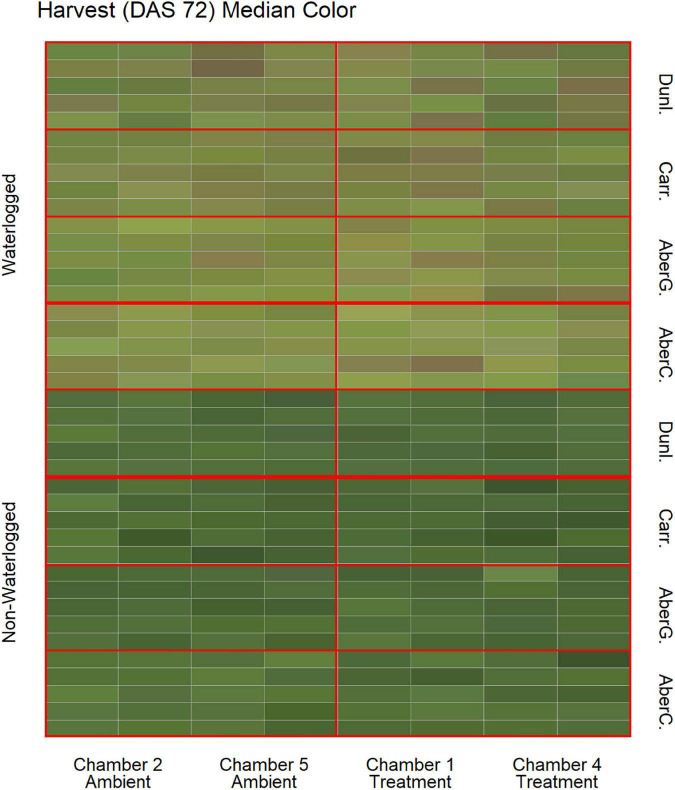
Median color (RGB) of the harvested material for all cores on DAS 72 as identified by the image analysis, sorted on water status, climate treatment and variety. Each cell represents the median true color of the harvested material of a plant. AberC, Aberchoice; AberG, Abergain; Carr, Carraig; Dunl, Dunluce.

**FIGURE 4:**
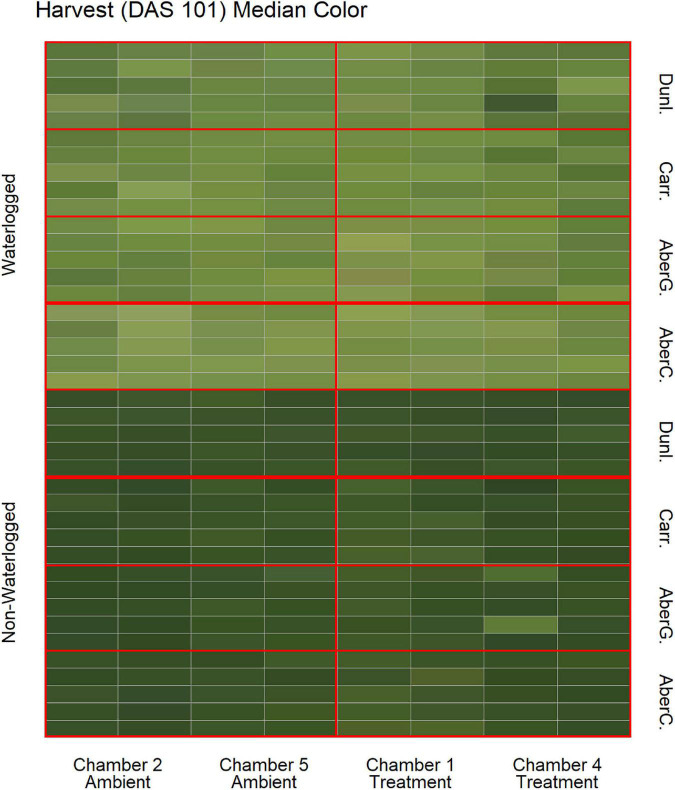
Median color (RGB) of the harvested material for all cores on DAS 101 as identified by the image analysis, sorted on water status, climate treatment and variety. Each cell represents the median true color of the harvested material of a plant. AberC, Aberchoice; AberG, Abergain; Carr, Carraig; Dunl, Dunluce.

To estimate the effects of long-term waterlogging on perennial ryegrass we grew 320 plants in fully atmospherically controlled growth chambers, simulating typical current climatic conditions in North-western Europe and conditions as predicted in 2050 according to RCP8.5 (550 ppm CO_2_ and a mean temperature increase of 2°C). Half of the plants were subjected to waterlogging 48 days after sowing (DAS) for one month. After waterlogging had begun, visible differences were observed between waterlogged and non-waterlogged plants at DAS 72 and continued to be visible at DAS 101 (Figure 1). The waterlogged plants by DAS 72 had stunted growth with dark brown leaf hues. The leaf morphology of the waterlogged plants also varied to the non-waterlogged plants, because leaf unfolding was disrupted, causing a concave and folded appearance of many leaves in waterlogged plants (Figure 2). By DAS 101 many plants had started to change in leaf color, with many leaves presenting light green shades. This contrasts with the non-waterlogged plants that had darker green shades and a lush appearance. Leaf morphology also differed at DAS 101 between waterlogged and non-waterlogged plants, where the leaves of the waterlogged plants remained concave and light green while the non-waterlogged plants had tall, wide and lush leaves. The median true colors of the harvests at DAS 72 (Figure 3) and DAS 101 (Figure 4) showed a substantial variation between plants of each water treatment and chamber condition, with darkening of leaves as the growth season progressed. No direct visual difference could be observed between the two climate treatments (ambient vs 2050 RCP8.5 scenario).

### Color quantification through RGB fusion

**FIGURE 5:**
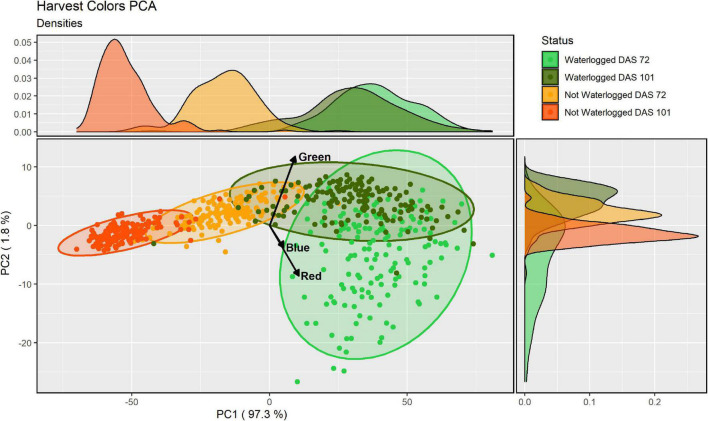
Principal component analysis (PCA)-analysis of the three RGB-hues of the harvested material for all cores, water status and harvest (DAS 72 and 101) as identified by the image analysis. Each ellipse represents a 95% confidence ellipse for each group. Side graphs shows the relative densities of each PCA axis and grouping, with the area under each curve representing the entire group distribution.

To quantify the visible differences between the waterlogged and non-waterlogged plants, all plant leaves were harvested at DAS 72 and again at DAS 101, photographed and subjected to a color analysis. The isolated RGB hues from the color analysis were further modeled using a PCA-method. We hypothesized that there would be measurable color differences depending on the water status, with the waterlogging contributing to lighter colors. We also expected color differences between harvest dates, with darker colors as the growth season progressed. The PCA of the three RGB hues isolated from the harvested material from DAS 72 and 101 identified that the greatest variation (97.3%) could be explained by the first PC axis (PC1) (Figure 5). All three RGB hues showed a positive increase with the first axis (Supplementary Table 1), illustrating that the axis describes a light beige to dark brown hue divergence (Supplementary Figure 1). To simplify this main leaf describing characteristics we classified the axis as overall color intensity. The negative values of PC1 are therefore darker intensities, while the positive values are lighter intensities. The other two minor axes PC2 (1.8%) and PC3 (0.9%) describe pure color hue gradients, a green-purple and an orange-blue hue divergence respectively. Kendall’s tau and Wilcoxon’s signed rank test showed that the harvested material from the waterlogged plants collected on DAS 72 were not correlated in color to the non-waterlogged plants of the same harvest and were overall lighter (*p* < 0.001) and more purple (*p* < 0.001) (Table 3). The waterlogged plants on DAS 101 were positively correlated in color intensity with the non-waterlogged plants of the same harvest while being darker (*p* < 0.001) and greener (*p* < 0.001). The harvested material from the waterlogged plants were positively correlated in color intensity from DAS 72 to 101 with the plants becoming darker (*p* < 0.001) and greener (*p* < 0.001) as they started to recover from the waterlogging. The non-waterlogged plants were positively correlated in color intensity between the harvests periods and became overall darker (*p* < 0.001) as the growth season progressed. See Supplementary Material for the mean differences in PC axes values between the harvests and water status groupings (Supplementary Table 2).

**TABLE 3:** Model statistics and significance levels for the comparison of PC axes values for the groupings harvested material (DAS 72 and 101) and water status of the cores in contrast to the other grouping.

PC axis	Water status		Harvest		Model statistics						
	Comparison		Comparison		Kendall’s tau Rank Correlation				Wilcoxon Signed-Rank Test		
					z	tau (τ)	*P*-value	Significance	V	*P*-value	Significance
PC1	Logged		DAS 72	DAS 101	9.979	0.532	<1 × 10^–10^	***	1,967	<1 × 10^–10^	***
	Normal		DAS 72	DAS 101	2.449	0.131	0.014	*	36	<1 × 10^–10^	***
PC2	Logged		DAS 72	DAS 101	1.757	0.094	0.079	NS	12,695	<1 × 10^–10^	***
	Normal		DAS 72	DAS 101	1.732	0.092	0.083	NS	445	<1 × 10^–10^	***
PC3	Logged		DAS 72	DAS 101	6.007	0.320	1.893 × 10^–9^	***	4,232	1.694 × 10^–4^	***
	Normal		DAS 72	DAS 101	5.495	0.293	3.907 × 10^–8^	***	12,875	<1 × 10^–10^	***
PC1	Logged	Normal	DAS 72		0.282	0.015	0.778	NS	0	<1 × 10^–10^	***
	Logged	Normal	DAS 101		3.332	0.178	8.629 × 10^–4^	***	2	<1 × 10^–10^	***
PC2	Logged	Normal	DAS 72		0.285	0.015	0.776	NS	12,053	<1 × 10^–10^	***
	Logged	Normal	DAS 101		−0.558	−0.030	0.577	NS	323	<1 × 10^–10^	***
PC3	Logged	Normal	DAS 72		1.374	0.073	0.170	NS	820	<1 × 10^–10^	***
	Logged	Normal	DAS 101		0.815	0.043	0.415	NS	9,506	1.765 × 10^–7^	***

The upper part of the table compares cores within the same water status between harvests. The lower part of the table compares between water status for the same harvest. DAS, days after sowing.

***p < 0.001.

**p < 0.01.

*p < 0.05.

^NS^p > 0.05.

### Phenotypic differentiation

To understand how phenotypic, environmental, genetic and temporal factors contribute to the quantified colors of the harvested leaf material a linear regression model was created and analyzed. The first PC axis (PC1), identified as color intensity, was modeled using three phenotypic variables, two environmental variables, one genetic variable and one temporal variable. The model was analyzed using Type II ANOVA and AIC to see the model performance after subsequently removing each variable individually. We hypothesized that the effects from the predictor variables would be physiologically connected and cause differences in color intensity. We expected that higher soil moisture would result in lighter colors and cause negative physiological effects on growth by lowering biomass and maximum height. We also expected that the climate change conditions would enhance growth through increased temperature and CO_2_, causing darker colors, along with darker colors as the growth season progressed, with variations between varieties caused by inherent genetic differences.

Phenotypically, harvested material from plants with lighter color intensities had significantly lower dried biomass (F_1,630_ = 232.44, *p* < 0.001), significantly lower maximum height (F_1,630_ = 57.92, *p* < 0.001), and significantly lower SPAD values (F_1,630_ = 250.01, *p* < 0.001) (Table 4 and Supplementary Figures 2–4). For the water status, harvested material from cores with lighter color intensities had significantly higher soil moisture (F_1,630_ = 174.67, *p* < 0.001) (Supplementary Figure 5). The harvested material from plants in chambers in predicted 2050 climate change conditions had significantly lighter intensities than harvested material from plants in ambient conditions (F_1,630_ = 19.20, *p* < 0.001) (Supplementary Figure 6).

**TABLE 4:** Model statistics and significance levels for the linear model in regards to the first PCA axis (Color Intensity) isolated from the color analysis of the harvested perennial ryegrass material.

Variable	Model statistics							
	Df	Sum Sq	RSS	AIC	Δ AIC	F-value	*P*-value	Significance
<none>			79,595	3,106.9				
Weight	1	29,367	108,962	3,305.9	−199.0	232.44	<1 × 10^–10^	***
Height	1	7,318	86,914	3,161.2	−54.3	57.92	<1 × 10^–10^	***
SPAD	1	31,587	111,182	3,318.8	−211.9	250.01	<1 × 10^–10^	***
Soil Moisture	1	22,068	101,663	3,261.5	−154.6	174.67	<1 × 10^–10^	***
Treatment	1	2,426	82,021	3,124.1	−17.2	19.20	1.379 × 10^–5^	***
Variety	3	12,664	92,259	3,195.4	−88.5	33.41	<1 × 10^–10^	***
Month	1	539	80,134	3,109.2	−2.3	4.27	0.039	*

Df, degrees of freedom; RSS, residual sum of squares.

Model Statistics: Anova (Type II) using the Drop1 function. Model performance: Adjusted R^2^ = 91.8%.

***p < 0.001.

**p < 0.01.

*p < 0.05.

^NS^p > 0.05.

Genetically, there were significant differences in color intensity between the varieties (F_3,630_ = 33.41, *p* < 0.001). *Aberchoice*, the only diploid variety, had the lightest color intensities, while the three tetraploid varieties, *Abergain, Dunluce*, and *Carraig* had darker intensities in that order, with *Carraig* having the darkest leaf color intensities (Supplementary Figure 7). The harvested material became darker overall as the season progressed (F_1,630_ = 4.27, *p* = 0.039), with the leaves in DAS 101 being significantly darker than DAS 72. The AIC revealed that the variables had a differential importance to the model performance. SPAD values (ΔAIC = −211.9), dried biomass (ΔAIC = −199.0), and soil moisture (ΔAIC = −154.7) were the most influential variables able to predict color intensity, while progression of the season (ΔAIC = −2.3), climate treatment (ΔAIC = −17.2) and maximum height (ΔAIC = −54.3) were the least influential. Overall, the linear regression model had a very high accuracy in predicting leaf color intensity, with an adjusted R^2^ of 91.8%. See Supplementary Material for the linear model estimates (Supplementary Table 3).

## Discussion

Our aim was to investigate the phenotypic effect of long-term waterlogging on perennial ryegrass. We found that long-term waterlogging reduced perennial ryegrass productivity and changed the plant phenotype. Waterlogged plants were lighter in color than controls, with younger waterlogged plants expressing purple hues, suggestive of anthocyanins. We also found that waterlogged plants had significantly lower dried biomass and maximum height, demonstrating that waterlogging reduced overall plant performance and yield. Our study has experimentally determined the influence of waterlogging and climate change upon plant performance, with the effects of waterlogging largely overshadowing the effects of increased temperature and elevated CO_2_. The imaging methods could be developed as remote sensed diagnostics tools in combination with drone-technology to determine the influence of waterlogging on plant health in field environments.

### Physiology and anthocyanins

Our results identified substantial variation in leaf coloration in perennial ryegrass caused by long-term waterlogging. This variation was mainly in color intensity, with waterlogging resulting in lighter color intensities. Previous studies have shown that waterlogging can reduce concentrations of plant pigments, mainly chlorophyll, resulting in lighter colors and reduced photosynthetic capability (e.g., Close and Davidson, 2003; Smethurst and Shabala, 2003; Pang et al., 2004; Li et al., 2011; Ou et al., 2011; Simova-Stoilova et al., 2012; Zhang et al., 2015; Barickman et al., 2019; Cotrozzi et al., 2021). Cotrozzi et al. (2021) found that waterlogging damaged the photosynthetic ability of durum wheat but that it depended on the duration, and that longer waterlogging was more detrimental. Li et al. (2011) found that waterlogging previous to anthesis could prevent photosynthetic damage in subsequent waterlogging events after anthesis, enhancing overall tolerance. Pang et al. (2004) found that barley varieties respond differently from waterlogging damage to photosystem II, and that recovery is at least partly genetically determined. We also observed significant variation in hue, with waterlogging resulting in red/blue (purple) shades. This was especially pronounced for the waterlogged plants in DAS 72, with large variation being observed between the plants, showing that varieties but also individual plants respond divergently to the same stimuli. These purple shades are likely caused by an accumulation of secondary anthocyanin metabolites, with multiple compounds having been identified in grasses (Clifford and Harborne, 1967; Fossen et al., 2002; Petrella et al., 2016). This suggests that individual plants respond differently to waterlogging by accumulating anthocyanins of varying degree. This is in agreement with previous research that has suggested that plants accumulate anthocyanins as a response to environmental stressors (Chalker-Scott, 1999), for example water availability as during drought (e.g., Close and Beadle, 2003; Kovinich et al., 2015; Li et al., 2018; Cirillo et al., 2021) and waterlogging (e.g., Close and Davidson, 2003; Smethurst and Shabala, 2003; Hussain et al., 2022). One of the main suggested benefits of increased anthocyanin accumulation is protection against DNA damaging UV-B radiation that can reduce photosynthetic capability (Teramura and Sullivan, 1994; Rozema et al., 1997; Hoch et al., 2001; Steyn et al., 2002). Anthocyanin accumulation has also been found as a response to phosphorus deficiency (e.g., Ulrychová and Sosnová, 1970; Shaikh et al., 2008; Sarker and Karmoker, 2011). This is a potential physiological cause of the observed purple shades in our waterlogged plants for two reasons. Firstly, phosphorus has been found to become soluble during excess water availability and migrate down the soil column (Sanyal and De Datta, 1991; Sinaj et al., 2002) and secondly, altered plant nutrient uptake during periods of excess water abundance might make the soil nutrients (e.g., phosphorus) temporarily unavailable (Elzenga and van Veen, 2010). These effects are expected to be especially pronounced for younger plants (Close and Beadle, 2003), as we indicated by our initial results, due to the undeveloped root system occupying only the upper part of the soil column. Additional root growth and reduction in soil moisture would allow the plants to reach the migrated phosphorus and start to recover via normalized nutrient levels, as observed with the reduction in purple hues from the waterlogging in DAS 101. Our results strengthen the consensus of recent studies that phenotypic color variations can effectively be quantified through image analysis and analyzed to detect physiological effects from waterlogging (e.g., de la Cruz Jiménez et al., 2017; Ventura et al., 2020), although detailed soil nutrient analysis would be needed to confirm our findings in connection to phosphorus availability. Future research is needed to validate the soil phosphorus availability in waterlogged scenarios in relation to anthocyanin accumulation in plant tissue and to what extent undeveloped root systems are influenced by this.

### Plant performance

Our results showed reductions in dried biomass and height in response to long-term waterlogging for all perennial ryegrass varieties. These reductions in plant growth likely stems from lower photosynthetic capability caused by a physiological reduction in plant pigments as identified from the SPAD values and color analysis, but also from hindrance to root development. Complex interactions between soil moisture and root proliferation patterns are likely one of the main drivers governing growth performance via soil nutrient absorption (Brugge, 1985; Setter and Belford, 1990; Xu et al., 2013; Medlyn et al., 2016; Cougnon et al., 2017). Previous studies have linked waterlogging to reductions in grass performance caused by altered root development (Malik et al., 2002; Ploschuk et al., 2017). This agrees with our findings, as we observed a clear reduction in root proliferation in the waterlogged cores (visual inspection, results not shown), with long-term waterlogging having previously been shown to reduce root mass in ryegrass (McFarlane et al., 2003). Another potential consequence is reduced root respiration caused by a reduction in soil oxygen levels, which has been shown to have multiple negative feedbacks on grass biomass accumulation and nutrients absorption (Trought and Drew, 1980; Dunbabin et al., 1997; Fukao et al., 2019).

There is also variation in waterlogging tolerance between grass species (e.g., Rubio et al., 1995; Rubio and Lavado, 1999; Xiao and David, 2019) and wheat cultivars (e.g., Ghobadi et al., 2017; Cotrozzi et al., 2021) based on local adaptation and specific genotypes. Genotype specific tolerance to waterlogging has been found between perennial ryegrass varieties (Liu and Jiang, 2015; Byrne et al., 2017). This was expanded upon by Pearson et al. (2011) that found multiple quantitative trait loci (QTLs) that code for morphological traits influencing the tolerance to waterlogging in perennial ryegrass. This is one likely explanation for the differential performance to waterlogging amongst our varieties, with the tetraploid varieties generally tolerating waterlogging better than the diploid variety. However, our study only included one diploid variety, introducing a potential risk for bias in evaluating the contribution of ploidy to waterlogging resilience. We did observe substantial variation between the tetraploid varieties, suggesting that other aspects of the genotype could influence the response to the environmental stressors. It is possible that the difference in performance is due to genotypic root development, which has been observed previously between perennial ryegrass varieties (Bonos et al., 2004; Wedderburn et al., 2010; Deru et al., 2014). Although, there is no clear connection between ploidy and stress tolerance to waterlogging or other environmental stressors (Yu et al., 2012; Kemesyte et al., 2017; Tozer et al., 2017; Lee et al., 2019). Future research would benefit from including a wide range of varieties of varying genotypes grown under combinations of environmental stressors coupled with detailed genomics analyses. This could reveal the genetic basis of stress tolerance, and potential genetic trade-offs that occur between phenotypic traits.

### Practical implications

We hypothesized that increases in temperature and CO_2_ (predicted 2050-levels) would enhance plant performance to climate change-induced waterlogging, but our results showed that perennial ryegrass responded with lighter leaf shades, suggesting reduced photosynthetic capability and reduced yields. This is in contrast to the theory that climate change will generally increase photosynthesis and productivity (Chen et al., 1996; Dusenge et al., 2019; Yiotis et al., 2021). Other studies have suggested that combined stressors brought by climate change could bring further negative effects than each stressor individually (Ahuja et al., 2010; Zandalinas et al., 2021). The developmental period of the grasses could be relevant here, with our study investigating the performance during the first few months including young plants. It is uncertain if these effects are specific to younger plants, or applicable to mature ones as well. Waterlogging has previously been shown to affect grain-crops differently depending on species and development, with wheat being able to sustain growth regardless of the period of waterlogging, while barley being disproportionately affected in later development (Ploschuk et al., 2020, 2021). Daepp et al. (2001) showed that the effects of elevated CO_2_ will depend on the developmental stage of the ryegrass, suggesting that some stages are more sensitive to than others. For grasses in general, elevated CO_2_ has been suggested to intensify the reproductive period from increases in NPP (Kurganskiy et al., 2021). Climate change will lead to multiple changes in the environment: increased temperature and CO_2_ may have positive effects by extending the growing season for certain species while simultaneously flooding or drought may have negative effects on overall plant growth (Richardson et al., 2013). To understand the effects of climate change we need to consider all environmental effects, their relative contribution to the overall impact and possible interactions to different developmental stages (Tubiello et al., 2007; Parmesan and Hanley, 2015; Gray and Brady, 2016; Zhou et al., 2020). In our case, the effect of waterlogging seems to largely overshadow the effects of increased temperature and elevated CO_2_.

To our knowledge this is the first study to investigate the combined effects of climate change (both temperature and CO_2_) and waterlogging experimentally in any plant. Previous studies have indicated that the interactive effects from elevated CO_2_ in isolation with waterlogging are inconclusive. Shimono et al. (2012) observed that soy bean (*Glycine max*) dry weight was significantly heavier during elevated CO_2_ (∼580–600 ppm), but did not see an overall interactive effect with waterlogging. Pérez-Jiménez et al. (2018) observed that the stress response of sweet cherry (*Prunus avium*) was significantly reduced after being waterlogged during elevated CO_2_ (800 ppm) compared to ambient levels, suggesting that elevated CO_2_ can reduce the waterlogging stress response. It is possible that the 200 ppm difference between these two studies is responsible for the difference in response, or that the study species inherently respond differently to the increase. One recent modeling-study has suggested that climate change will reduce waterlogging stress in barley (Liu et al., 2021), but not enough to compensate the reduction in yield (∼35% on average) due to high temperatures stress and that the development of resilient varieties to waterlogging will be required. However, increases in flooding due to climate change would cause more severe impacts than from current precipitation regimes (Mirza, 2011; Iglesias et al., 2012). The decrease in overall dried biomass for all varieties as a consequence of waterlogging predicted by our study has implications for global food security. A reduction in overall plant yield due to poor plant growth from flooding would potentially cause increases in fodder prices with follow-up consequences to all agricultural sectors and industries relying on fodder from pasture lands (Hazell and Wood, 2008; Kipling et al., 2016b; Manik et al., 2019). Early detection of waterlogging and identification of varieties resistant to waterlogging, will likely be important to employ mitigation strategies that could minimize reductions in plant health and production yield in pasture lands. Areas prone to current waterlogging are likely to experience increased frequency with climate change, with the utilization of appropriate drainage systems (natural or artificial) have previously been shown to be effective (Singh, 2017; Manik et al., 2019). The identification and breeding of plant lineages resilient to waterlogging damage is likely the most efficient (and cost-effective) approach of mitigating reductions in yield and performance and will be fundamental in a climate change future (Boru et al., 2001; Kole et al., 2015; Rivero et al., 2022).

We demonstrate that image analysis approaches can be used as diagnostic tools to investigate plant performance reductions caused by waterlogging. While most field monitoring would be time consuming and most satellite-based remote sensing products would be low resolution for this type of analysis other more navigable high-resolution options are available, for example drones (Bansod et al., 2017; Cracknell, 2018; Simic Milas et al., 2018). Drones have been shown to be a useful and cost-effective tool for agricultural surveying (Tripicchio et al., 2015; Puri et al., 2017; Kulbacki et al., 2018) and plant ecological investigation (Cruzan et al., 2016; Tay et al., 2018; Zellweger et al., 2019; Sun et al., 2021). The main benefits comes from the use of high-resolution multi- and hyperspectral cameras which capture a wide-range of light wavelengths used to infer plant physiological parameters (e.g., Li et al., 2020; Tao et al., 2020; Papp et al., 2021). Our results showed that differences in color intensity could be observed between perennial ryegrass varieties, suggesting that the imaging analysis method could be developed further to identify closely related varieties or perhaps different grass species using remote-sensed color distributions. Recent studies have also shown the usefulness of drones to monitor the effects of waterlogging on agricultural systems (e.g., Boiarskii et al., 2019; Den Besten et al., 2021; León-Rueda et al., 2021), illustrating that our imaging approaches could be adapted to work as diagnostic tools with drone-technology. Applications using integrated monitoring of plant health will become increasingly important as climate change-induced extreme weather events become more prevalent.

## Data availability statement

The data supporting the findings of this study is now publicly available in the general-purpose open-access repository Zenodo developed by the European OpenAIRE program and operated by CERN (doi: 10.5281/zenodo.6334191).

## Author contributions

CF: design of the research, performance of the research, data analysis, collection or interpretation, and writing the manuscript. GX-S, MO, and YB: performance of the research and data analysis, collection, or interpretation. RM and JY: design of the research, data analysis, collection or interpretation, and writing the manuscript. All authors contributed to the article and approved the submitted version.
