# Supplementary material for: Phenotypic variation from waterlogging in multiple perennial ryegrass varieties under climate change conditions
Source: Front Plant Sci. 2022 Aug 4;13:954478. doi: 10.3389/fpls.2022.954478 (PMC9387306; doi:10.3389/fpls.2022.954478)
Supplement: Supplementary file 1 [file Data_Sheet_1.pdf]

Supplementary Material

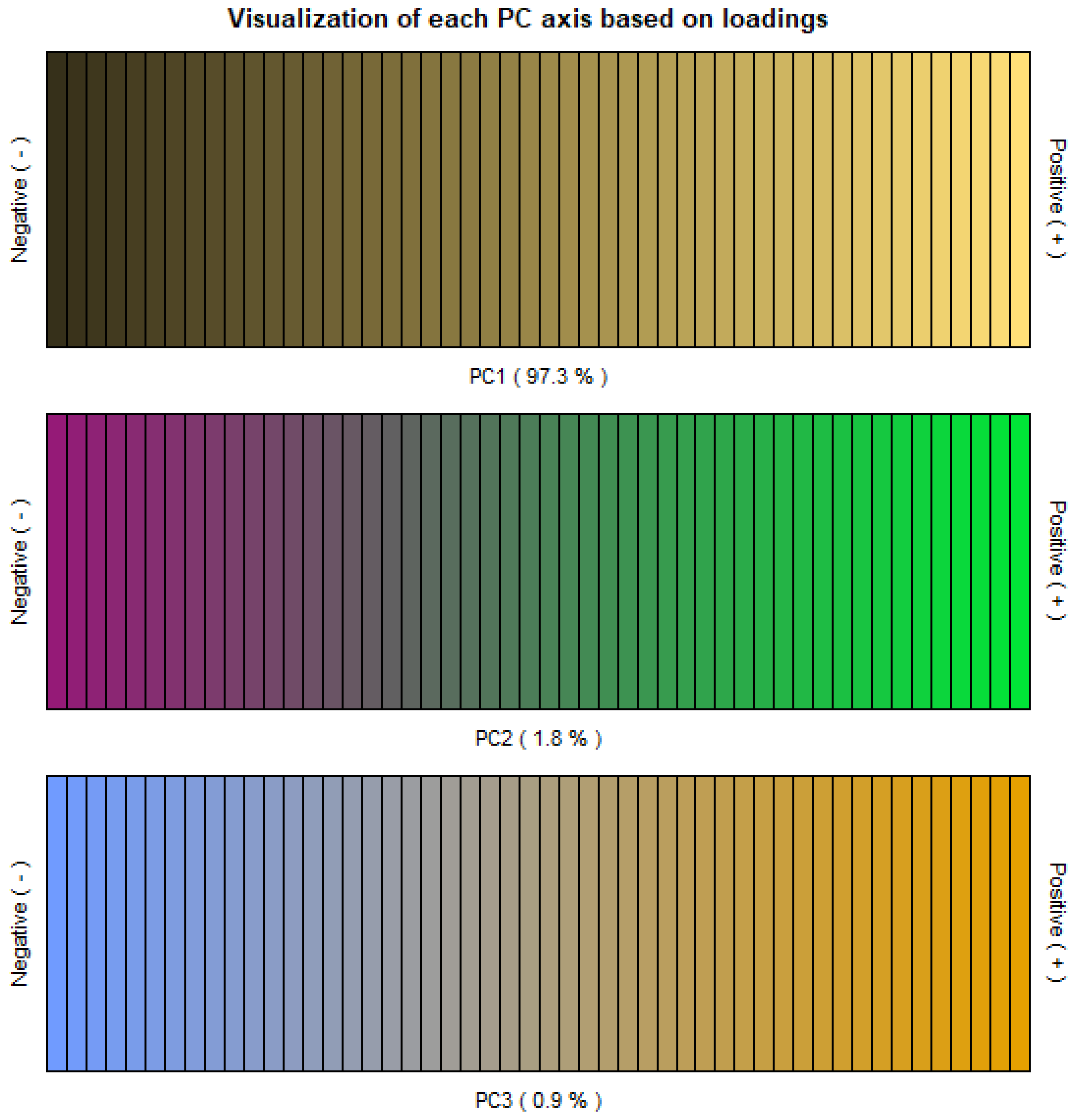

Supplementary Figure 1. Visualization of the color spectrum of each PC axis based on the loadings from each RGB hue from the image analysis.

Supplementary Table 1

Axes loadings and model variation of the Principal Component Analysis (PCA) isolated and modelled from the color analysis of the harvested perennial ryegrass material.

**Axis**	**Loadings Color Hue**			**Variation**	
	Red	Green	Blue	SD	Variance (%)
PC1	0.707	0.621	0.337	39.222	97.3%
PC2	-0.570	0.783	-0.248	5.338	1.8%
PC3	0.419	0.016	-0.908	3.761	0.9%

Supplementary Table 2

Mean value and standard deviation for each grouping of water status and harvests per PC axis in the Principal Component Analysis (PCA). **Abbreviation: DAS** - Days after Sowing.

**Water Status**	**Harvest**	**PC1 (97.3 %)**		**PC2 (1.8 %)**		**PC3 (0.9 %)**	
		Mean	SD	Mean	SD	Mean	SD
Logged	DAS 72	38.67	14.11	-4.98	7.22	1.30	3.57
	DAS 101	29.92	17.96	4.07	2.27	0.04	3.74
Normal	DAS 72	-16.27	11.66	2.19	2.10	-3.33	2.83
	DAS 101	-52.32	9.90	-1.28	1.57	1.99	2.29

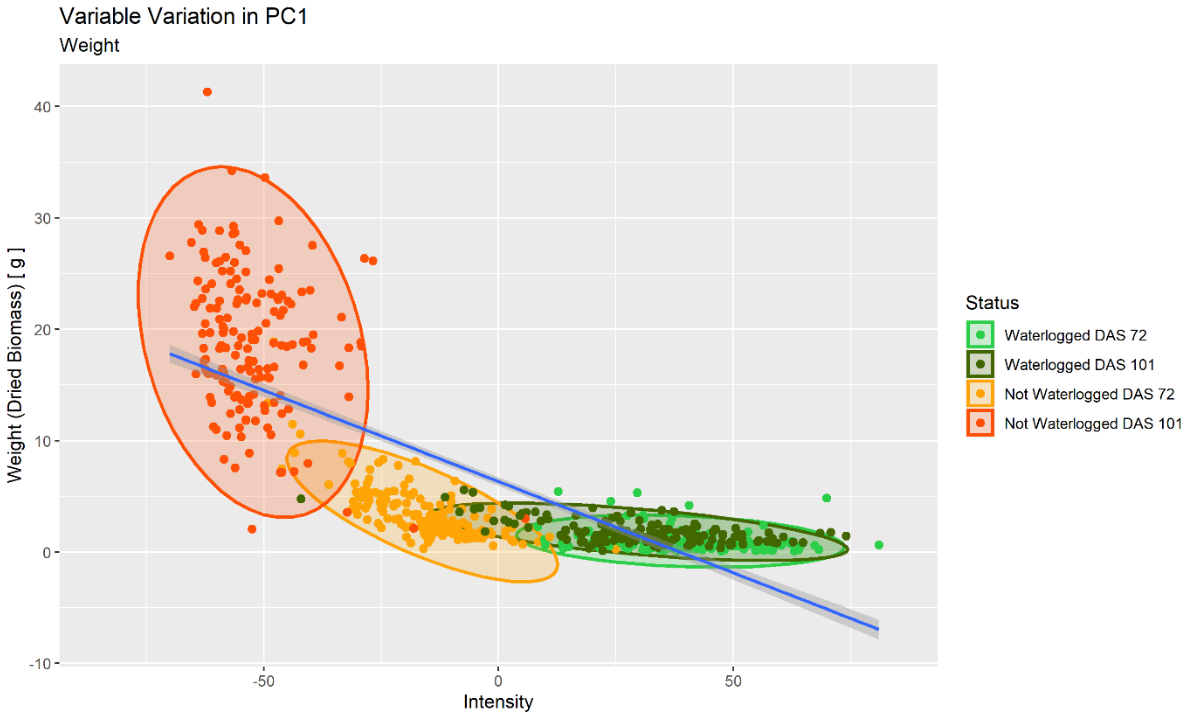

Supplementary Figure 2. Relationship between dried biomass and color intensity (PC1) for all harvested cores, water status and harvest (DAS 72 and 101) as identified by the image analysis. Each ellipse represents a 95% confidence ellipse for each group. The trend line is a smoother based on linear regression (y~x) with 95% confidence intervals.

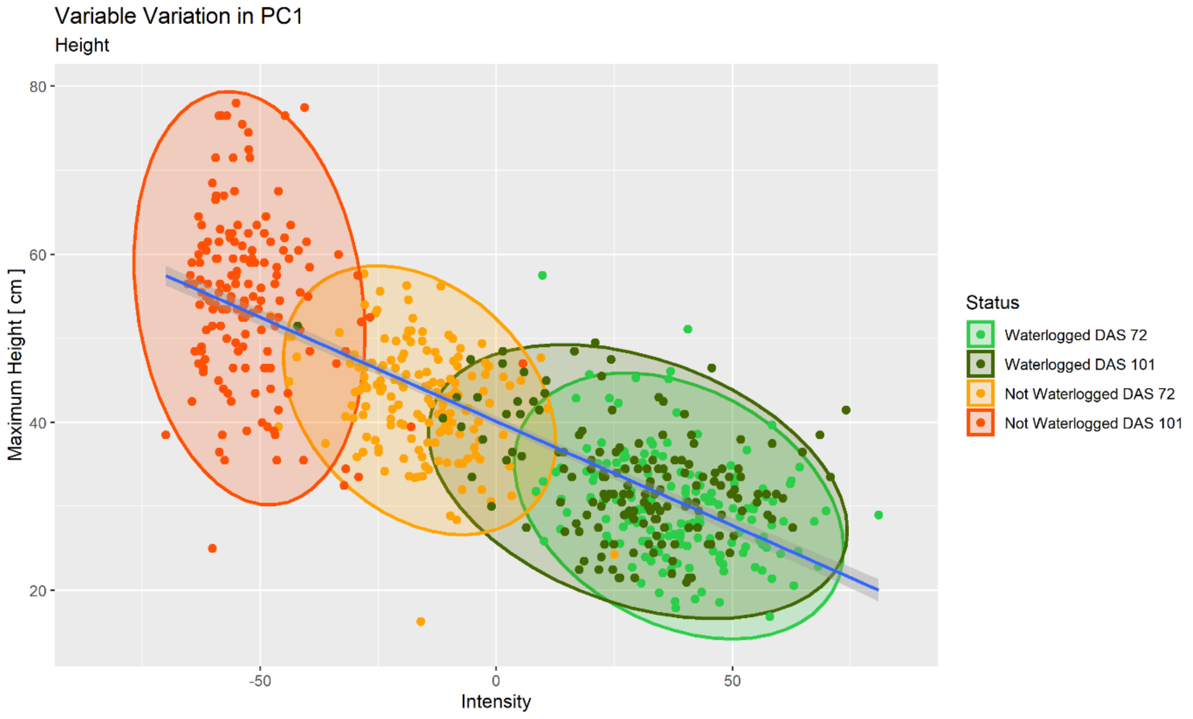

Supplementary Figure 3. Relationship between maximum height and color intensity (PC1) for all harvested cores, water status and harvest (DAS 72 and 101) as identified by the image analysis. Each ellipse represents a 95% confidence ellipse for each group. The trend line is a smoother based on linear regression (y~x) with 95% confidence intervals.

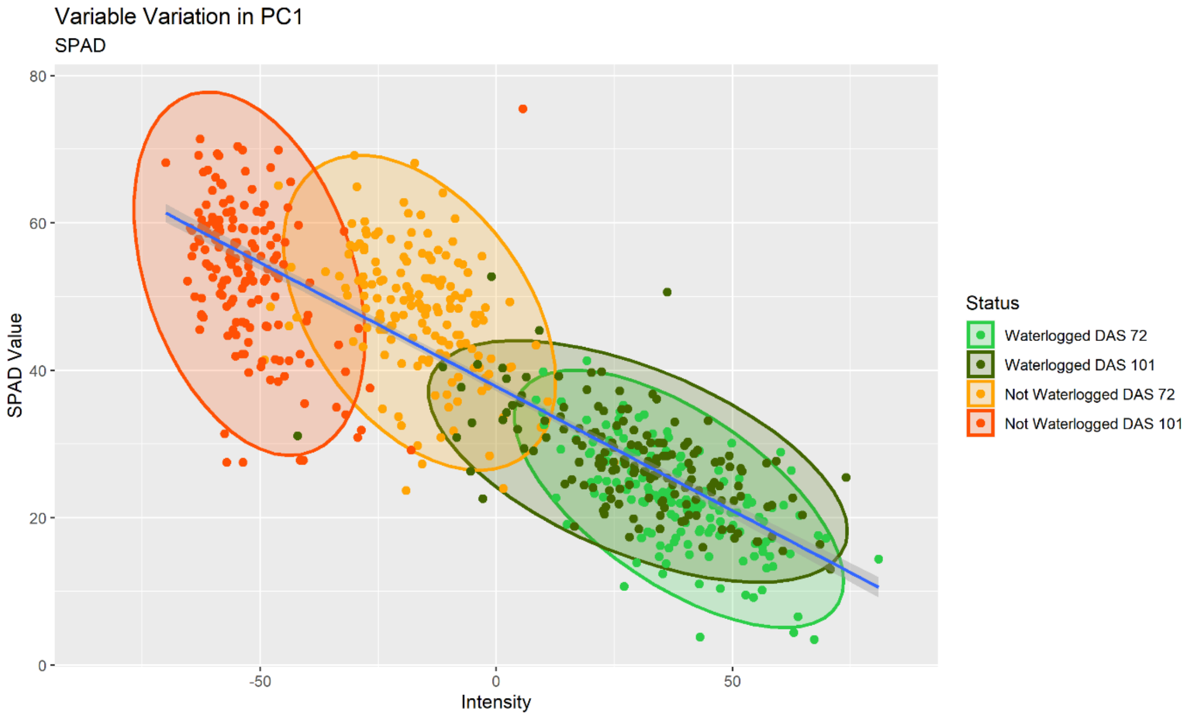

Supplementary Figure 4. Relationship between SPAD values and color intensity (PC1) for all harvested cores, water status and harvest (DAS 72 and 101) as identified by the image analysis. Each ellipse represents a 95% confidence ellipse for each group. The trend line is a smoother based on linear regression (y~x) with 95% confidence intervals.

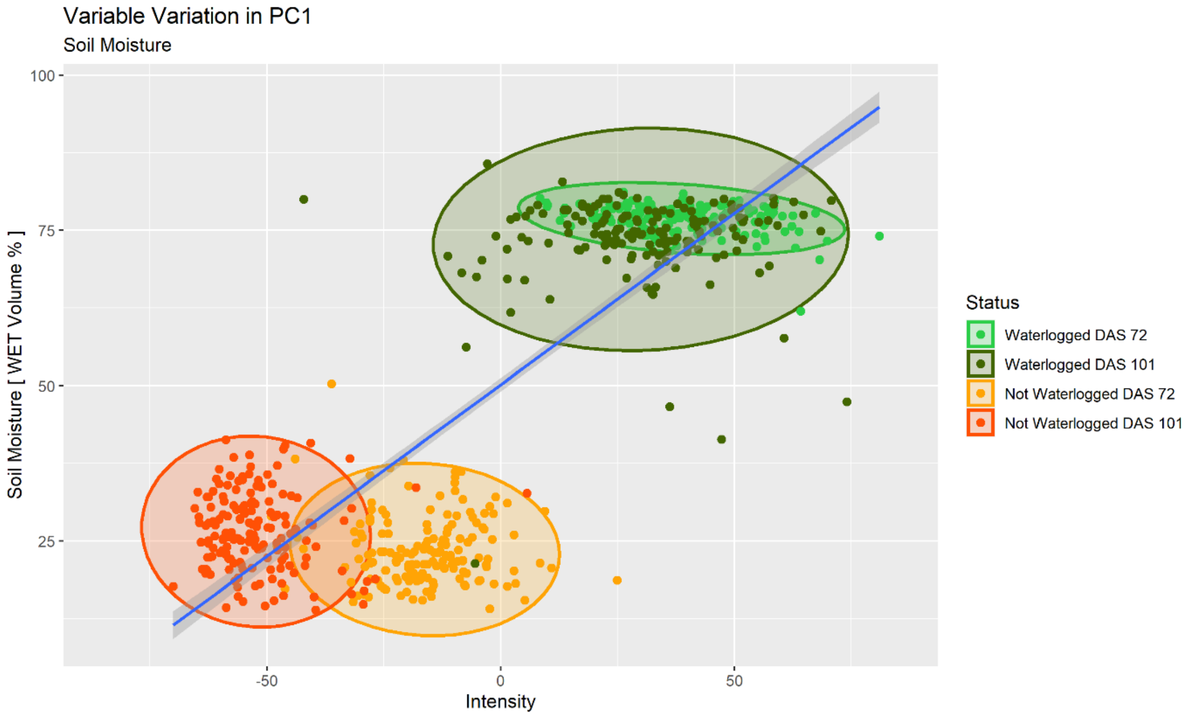

Supplementary Figure 5. Relationship between soil moisture and color intensity (PC1) for all harvested cores, water status and harvest (DAS 72 and 101) as identified by the image analysis. Each ellipse represents a 95% confidence ellipse for each group. The trend line is a smoother based on linear regression (y~x) with 95% confidence intervals.

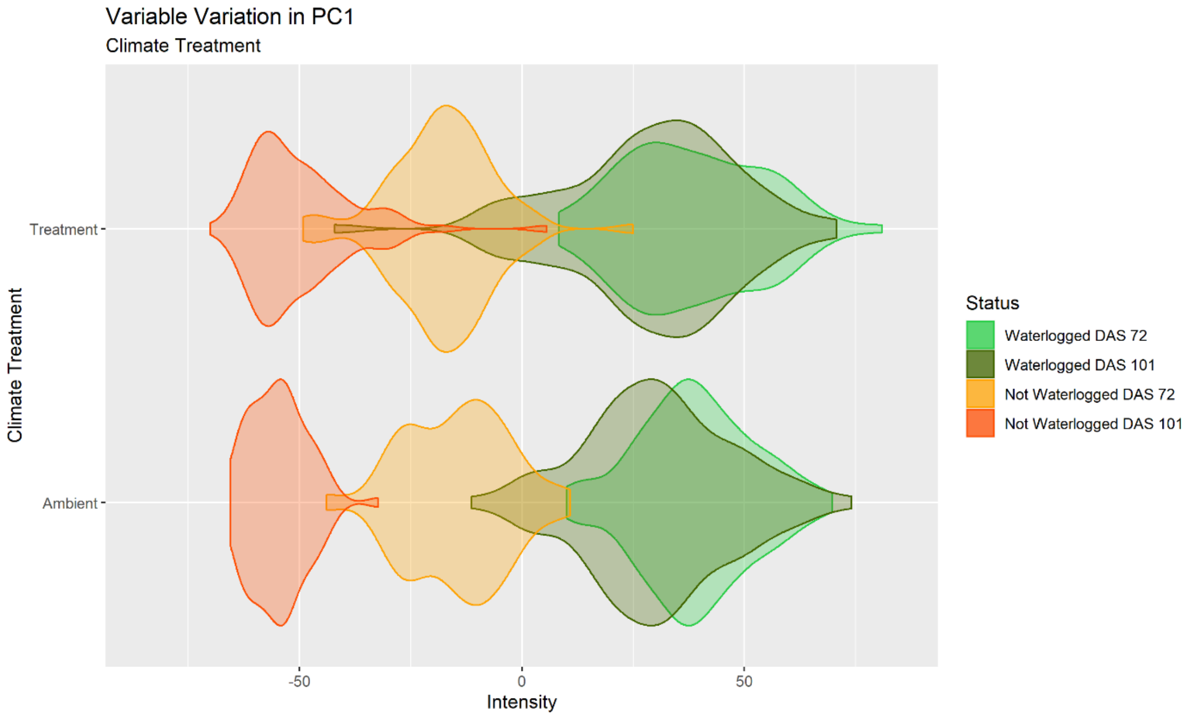

Supplementary Figure 6. Violin graphs describing the relationship between climate treatment and color intensity (PC1) for all harvested cores, water status and harvest (DAS 72 and 101) as identified by the image analysis.

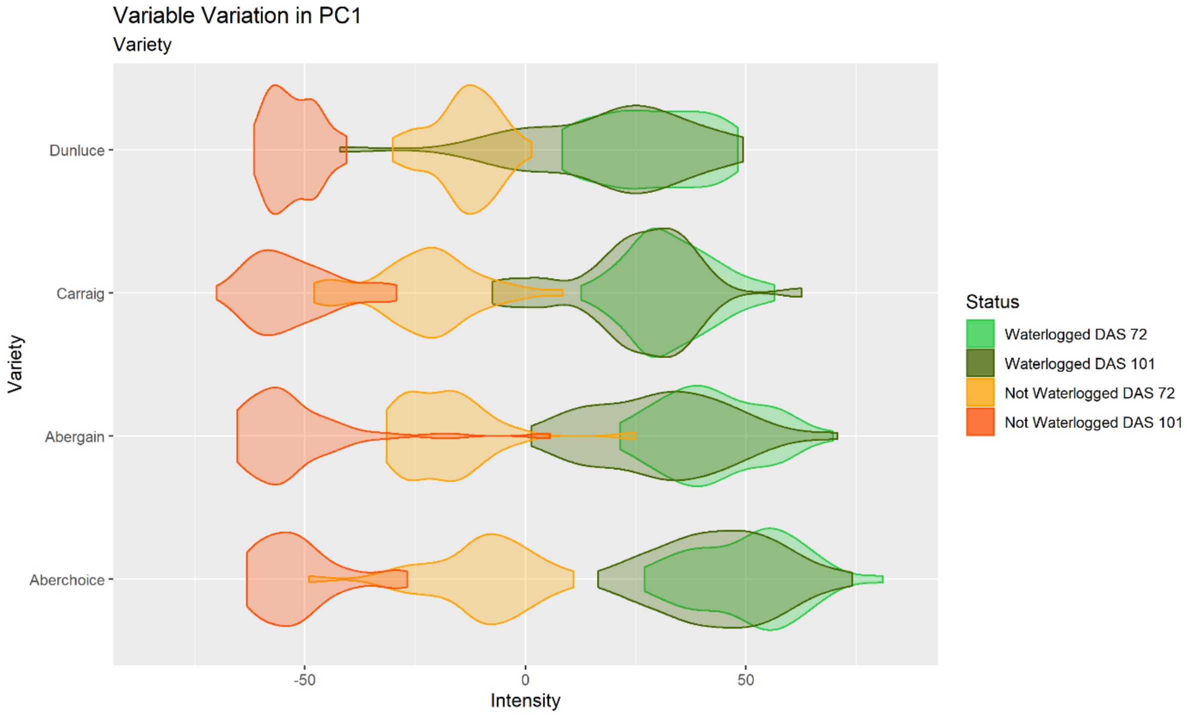

Supplementary Figure 7. Violin graphs describing the relationship between varieties and color intensity (PC1) for all harvested cores, water status and harvest (DAS 72 and 101) as identified by the image analysis.

Supplementary Table 3

Model statistics for the variables in the linear regression model in regard to the first PCA axis.
Intercept: Treatment - Ambient, **Variety** - Aberchoice, **Harvest** - DAS 101. Extension of **Table 4.**
Abbreviation: DAS - Days after Sowing

**Variable**		**Model Statistics**		
Main variable	Sub-factor	Estimate	Std. Error	t - value
Intercept		42.540	4.385	9.701
Weight		-1.481	0.097	-15.246
Height		-0.483	0.063	-7.611
SPAD		-0.850	0.054	-15.812
Soil Moisture		0.446	0.034	13.216
Treatment	eCO_2 + 2°C	3.980	0.908	4.382
Variety	Abergain	-6.793	1.273	-5.336
	Carraig	-11.429	1.288	-8.877
	Dunluce	-10.805	1.297	-8.329
Harvest	DAS 72	2.377	1.151	2.065
